# The Interaction Between Intracellular Energy Metabolism and Signaling Pathways During Osteogenesis

**DOI:** 10.3389/fmolb.2021.807487

**Published:** 2022-01-28

**Authors:** Jiapeng Ye, Jirimutu Xiao, Jianwei Wang, Yong Ma, Yafeng Zhang, Qiang Zhang, Zongrui Zhang, Heng Yin

**Affiliations:** ^1^ Wuxi TCM Hospital Affiliated to Nanjing University of Chinese Medicine, Nanjing University of Traditional Chinese Medicine, Nanjing, China; ^2^ Mongolian Medicine College, Inner Mongolia Medical University, Hohhot, China; ^3^ Department of Orthopedics and Traumatology, Wuxi TCM Hospital Affiliated to Nanjing University of Chinese Medicine, Wuxi, China

**Keywords:** osteoporosis, osteoblast differentiation, energy metabolism, acetylation, O-GlcNAc

## Abstract

Osteoblasts primarily mediate bone formation, maintain bone structure, and regulate bone mineralization, which plays an important role in bone remodeling. In the past decades, the roles of cytokines, signaling proteins, and transcription factors in osteoblasts have been widely studied. However, whether the energy metabolism of cells can be regulated by these factors to affect the differentiation and functioning of osteoblasts has not been explored in depth. In addition, the signaling and energy metabolism pathways are not independent but closely connected. Although energy metabolism is mediated by signaling pathways, some intermediates of energy metabolism can participate in protein post-translational modification. The content of intermediates, such as acetyl coenzyme A (acetyl CoA) and uridine diphosphate N-acetylglucosamine (UDP-N-acetylglucosamine), determines the degree of acetylation and glycosylation in terms of the availability of energy-producing substrates. The utilization of intracellular metabolic resources and cell survival, proliferation, and differentiation are all related to the integration of metabolic and signaling pathways. In this paper, the interaction between the energy metabolism pathway and osteogenic signaling pathway in osteoblasts and bone marrow mesenchymal stem cells (BMSCs) will be discussed.

## Introduction

Due to our in-depth study and rapid understanding of bioenergy in cells, new therapeutic targets have emerged, and cell metabolism has become a hot spot in the study of chronic diseases. Similarly, the changes in the energy production process and its regulatory mechanisms in bone cells during bone repair and regeneration have been widely studied. Osteoblasts produced by bone marrow mesenchymal stem cells (BMSCs) in the process of bone formation, remodeling, and fracture healing are the main bone-forming cells in human skeletal homeostasis (Long, 2012). Osteoblasts have special choices for energy utilization and metabolic substrate selection due to their responsibility for the synthesis, secretion, and mineralization of the bone matrix. Some diseases in which energy availability is disordered, such as diabetes, anorexia nervosa, and aging, eventually lead to osteopenia and osteoporotic fractures. The bioenergetics and metabolic processes of osteoblasts are poorly understood; therefore, the sources and pathways of adenosine triphosphate (ATP) production by these cells need to be studied further, including the metabolic changes in glucose, fatty acids, and amino acids in cells. Intracellular metabolism provides enough energy and corresponding substrates for biosynthesis. These enzymes and metabolites participate in signal transduction and gene transcription regulation (Rupprecht et al., 2014). Cell differentiation lineage and proliferation and the execution of corresponding functions can cause changes in energy metabolism, which can also be affected and regulated by these processes (Shyh-Chang and Daley, 2015). Different energy metabolism phenotypes are exhibited in different cells according to their function and environment. In this paper, we hope to clarify the relationship between energy metabolism and the osteoblast signaling pathway and provide a new idea for the development of bone anabolic drugs. A new method for the treatment of osteoporosis can be created by changing the energy metabolism of osteoblasts to improve osteogenesis.

## Intracellular Energy Metabolism During Osteogenesis

### The Glucose Metabolic Pathway

Several proteins related to glucose transportation (GLUT), such as GLUT1, GLUT3, and GLUT4, can be expressed on the surface of osteoblasts. These proteins can also regulate to the functioning of osteoblasts (Lee et al., 2017). The processes involved in the glycolysis pathway are as follows. In the cytoplasm, glucose is metabolized by a series of glycolytic enzymes, such as hexokinase (HK), phosphoglucose isomerase, aldolase, and pyruvate kinase, and converted into pyruvate. Under anaerobic conditions, pyruvate is further converted into lactic acid and only a small amount of ATP is produced to provide energy for the cells. This process is inhibited under aerobic conditions. Pyruvate enters the mitochondria to participate in the tricarboxylic acid cycle and is completely oxidized, resulting in the formation of a large quantity of ATP to provide sufficient energy. Glucose-6-phosphate (G6P), an intermediate of glycolysis, can also be metabolized through the pentose phosphate pathway (PPP) to produce 5-phosphate ribose, which is required for nucleic acid synthesis, and nicotinamide adenine dinucleotide phosphate to maintain redox stability. In addition, G6P is converted to fructose-6-phosphate by phosphohexose isomerase, which can be metabolized via the hexosamine biosynthesis pathway to produce uridine diphosphate N-acetylglucosamine (UDP-GlcNAc) to participate in protein glycosylation (Mulukutla et al., 2016). Glucose is an important energy-producing substance. Studies on the bioenergy of cells have shown that the levels of glycolysis and oxidative phosphorylation (OXPHOS) in differentiated osteoblasts are higher than in undifferentiated cells, but the oxygen consumption rate (OCR) to extracellular acidification rate ratio gradually decreases, which indicates that differentiated cells are more capable of glycolysis than undifferentiated cells (Guntur et al., 2014). This is known as aerobic glycolysis, which is similar to the Warburg effect in cancer cells (Li et al., 2018). An untargeted metabolomics study in primary mouse BMSCs revealed metabolic changes during osteoblast differentiation, and intracellular metabolite profiles similarly revealed that OXPHOS is a major source of ATP during the early stages of differentiation, with the energy source of mature osteoblasts being dependent on glycolysis (Misra et al., 2021). Meyer et al. (2018) analyzed the proliferation and differentiation of human adipose-derived mesenchymal stromal cells (adMCS). The contents of the glycolysis marker enzyme, phosphofructokinase (PFK), and the PPP marker enzyme, 6-phosphate glucose dehydrogenase, increased under the conditions of undifferentiation and osteogenic induction, but the glycolytic ability decreased with a decrease in cell proliferation in the process of adipocyte differentiation, e.g., the ability of glyceraldehyde 3-phosphate dehydrogenase decreased, mitochondrial enzyme activity increased, and OXPHOS was enhanced, which indicates that the energy metabolism of the adMCS is adapted to the direction of the specific cell differentiation during osteogenic and adipogenic differentiation. Therefore, once glucose enters the osteoblasts, its catabolic pathway is highly dependent on the stage and direction of differentiation. Yang et al. (2020) believed that aerobic glycolysis is necessary because it can produce ATP at a faster rate, provide intermediate metabolites that are used to synthesize matrix proteins, and secrete citrate, which is an important component of the apatite nanocrystalline structure in bone. Karner et al. (2016) believed that this metabolic mode regulates the differentiation and function of osteoblasts through epigenetics, which will be explained in detail below. However, Shum et al. (2016) found that mitochondrial OXPHOS is increased in during the osteogenic differentiation of human mesenchymal stem cells (hMCS) and the glycolytic level of hMCS did not change significantly when compared with undifferentiated cells. These phenomena indicate that cells have different energy metabolic preferences at different stages of osteogenic differentiation and maturation.

### Amino Acid Metabolism

Amino acids are divided into ketogenic or glycogenic amino acids and are the basic raw materials needed for osteoblasts to synthesize protein or produce ATP. Notably, ketogenic amino acids are converted into acetyl CoA or acetoacetate *in vivo*, while glycogenic amino acids are metabolized into pyruvic acid or intermediates of the tricarboxylic acid (TCA) cycle. Early studies showed that cyclic adenosine monophosphate (cAMP), thyroid hormones, insulin, and insulin-like growth factor 1 (IGF1) regulate transport in osteoblasts (Dirckx et al., 2019). SLC1A5, which mainly transports glutamine and asparagine, has been found to be a key amino acid transporter involved in protein synthesis and osteoblast differentiation during skeletal development (Sharma et al., 2021). General control nonderepressible 2 (GCN2) regulates the proliferation of mice skeletal stem/progenitor cells by increasing the transcription effector activating transcription factor 4 to increase the ingestion of amino acids, which is conducive to maintaining bone homeostasis (Hu et al., 2020). Glutamine catabolism has been widely studied in cancer. It is involved in reprogramming energy metabolism, providing nitrogen sources, and signal transduction (Bott et al., 2019). Glutamine is converted into glutamic acid by glutaminase (GLS) and then converted to α-ketoglutarate to enter the TCA cycle. Therefore, glutamine becomes an ideal bioenergy substrate for cells that are highly dependent on glycolysis and provides an intermediary for active biosynthesis (DeBerardinis et al., 2007; Fan et al., 2013).

### Fatty Acid Metabolism

Fatty acids are activated in the cell matrix to form acyl CoA, which is then transported into the mitochondrial matrix by carnitine palmitoyltransferase (CPT1 and CPT2) in the inner and outer membranes of the mitochondria and undergoes *ß*-oxidation to produce ATP. The energy produced by fatty acid oxidation far exceeds that provided by glucose or amino acid metabolism. In 1987, Adamek et al. (1987) found that fatty acids also play an important role in providing energy for bone tissue *in vivo* and cells *in vitro*. Osteoblasts can oxidize fatty acids *in vitro*, and the utilization of fatty acids is controlled by hormones (Suchacki et al., 2016). Etomoxir (a fatty acid oxidation inhibitor) has adverse effects on osteoblast mineralization and cell growth *in vitro* (Rendina-Ruedy et al., 2017). These findings suggest that the utilization of fatty acids is the key to maintaining the normal physiological functioning of bone.

## The Effect of the Intracellular Signaling Pathway on Energy Metabolism During Osteogenesis

### The Effect of Estrogen-Related Receptor α on Energy Metabolism

The dynamic expression pattern of the age-related estrogen-related receptor α (ERR α) of cells is related to the differentiation of BMSCs into osteoblasts, and the content of ERRα is significantly reduced in aged rats (Huang et al., 2017; Wang et al., 2019). At the cellular level, the protein expression of ERRα reaches a peak in the early stage of osteoblast differentiation and decreases in the mineralization stage. However, the level of ERRα messenger RNA (mRNA) remains stable, which indicates that ERRα is degraded after osteoblast maturation and regulates the degree of osteoblast differentiation in a time-dependent pattern (Gallet et al., 2013). The aging of an organism often reveals dysfunction of the cell mitochondria. The synergistic activation of ERRα and peroxisome proliferator activated receptor γ coactivator1 α regulates mitochondrial biogenesis through fatty acid oxidation and energy consumption related to reactive oxygen species (ROS) production, but its effect on osteoblasts needs further study (Bonnelye and Aubin, 2013; Luo et al., 2017). Lin et al. (2017) found that estradiol-stimulating human osteoblasts promote the ERRα translocation into the nucleus to induce the expression of respiratory chain complexes and cytochrome c oxidase genes because there are four estrogen-receptor complex binding sites in the 5’ promoter region of mitochondrial CoxI gene. At the same time, it can stimulate the transfer of ERRα from the cytoplasm to the mitochondria. The study indicated that the estradiol/ERRα signal axis promotes osteoblast maturation and enhances osteoblast anabolism by inducing the expression of complex genes of the chromosomes and mitochondria and upregulating the mitochondrial bioenergy system. Wu et al. (2020) supported these findings. Studies on fracture healing found that the bone mass around a bone defect increased over time. The level of ERRα was specifically upregulated and translocated to the mitochondria, and the expression of Cox I and CoxII mRNA related to mitochondrial energy production and alkaline phosphatase (ALP), runt-related transcription factor 2 (Runx2), and osteocalcin (OCN) mRNA related to osteogenesis were upregulated. ERRα regulates the expression of GLS by directly binding to the promoter of the GLS gene, leading to mitochondrial glutamine supplementation. Resuming the expression of ERRα and GLS can improve the osteogenic differentiation ability of MSCs to resist low bone mass because aging attenuates the effect of the ERRα/GLS pathway. mTOR is a major biosynthetic regulator that can affect the transcriptional activity of ERRα to regulate the ERRα/GLS signaling pathway (Huang et al., 2017). Therefore, mTOR may become a target of some drugs to treat senile osteoporosis. Studies on osteoblasts and osteocytes in mice in which CPT2 expression has been specifically disrupted showed only transient trabecular defects in the male mutants. This phenomenon is a result of the rapid increase in the glucose uptake and consumption rate in bone tissue, and the metabolic flexibility of female mutants is lower than male mutants and many defects were observed in the bone structure (Kim et al., 2017). Estrogen is beneficial to the utilization of fatty acids but not to the metabolism of glucose in many tissues (Campbell and Febbraio, 2001), and it may lead to sex dimorphism of the bone phenotype in this model. Therefore, it can be inferred that estrogen and ERRα have certain effects on fatty acid metabolism during osteogenesis. In addition, the dynamic changes of ERRα in osteogenic differentiation and the specific mechanism of cellular metabolism need to be studied further.

### The Effect of Parathyroid Hormone on Energy Metabolism

Parathyroid hormone (PTH) can increase the number of early stage osteoblast lineage cells, accelerate their differentiation into osteoblasts, and inhibit adipogenic differentiation (Balani et al., 2017). Esen et al. (2015) found that PTH increased glucose absorption and oxygen consumption during the induction of osteoblast differentiation. Interestingly, the metabolites of glycolysis entering the TCA cycle were inhibited. However, PTH induced lactate production, which may be achieved by increasing the abundance of lactate dehydrogenase A (LDHA) because knockout of this enzyme gene eliminated the influence of PTH on glucose catabolism. In order to prove that PTH can promote bone biosynthesis through the metabolic pathway *in vivo*, dichloroacetic acid (DCA), an inhibitor of pyruvate dehydrogenase kinase (PDH), was used to increase pyruvate entering the TCA cycle and decrease aerobic glycolysis, which significantly inhibited the bone synthesizing function of PTH. The IGF1 signal induced by PTH triggers the activation of the mTORC2 complex, which is an essential factor for bone metabolism (Wang et al., 2007). Studies have shown that the mTORC2 complex participates in bone growth and induces aerobic glycolysis (Esen et al., 2013; Chen et al., 2015). However, Esen demonstrated that PTH inhibits glucose oxidation in the TCA cycle and increases oxygen consumption. The most likely mechanism for these phenomena is that PTH facilitates the oxidative metabolism of other energy-producing substrates to produce ATP. Karner et al. (2015) and Frey et al. (2015) demonstrated that glutamine and fatty acids are significant energy substrates for osteoblasts. The PTH signal is considered a regulator of fatty acid utilization, which can induce adipolysis of adipocytes (Larsson et al., 2016). It has been proved *in vivo* that PTH can inhibit osteoporosis by promoting the transition to the osteogenic lineage and reducing the formation of bone marrow adipose tissue and *in vitro* that fatty acids transfer from adipocytes to osteoblast lineage cells after lipolysis (Fan et al., 2017; Maridas et al., 2019). Fatty acid oxidation is particularly sensitive to the cAMP/protein kinase A (PKA) signaling pathway, which is activated by PTH, thus inhibiting fatty acid *ß* oxidation and damaging osteoblast differentiation *in vitro* (Gerhart-Hines et al., 2011; Frey et al., 2015).

### The Effect of the Wnt Signaling Pathway on Energy Metabolism

Previous research has shown that the Wnt signaling pathway boosts the biological behavior of osteoblasts and osteoblast differentiation of BMSCs by increasing the number of cells and protein synthesis activity and directly recombines osteoblast metabolism by stimulating aerobic glycolysis, fatty acid oxidation, and glutamine catabolism (Karner and Long, 2017). ST2 cells (bone marrow stromal cells of model mice) cultured *in vitro* showed that Wnt3a and Wnt10b increase a large number of key enzymes, e.g., HK2, phosphate fructose kinase 1(PFK1), LDHA, and fructose-2,6-biphosphatase 3(PFKFB3), involved in glycolysis through the Wnt coreceptor low-density lipoprotein receptor-related protein 5 (LRP5), and glucose transport also increases by stimulating the expression of GLUT1. Glycolysis enzymes are regulated and controlled by the activation of the mTORC2 and Akt rather than the *ß*-catenin and Glycogen synthase kinase-3 β(GSK3-β) signaling pathways because the inhibition of these effectors does not affect glucose consumption. However, Wnt3a activates mTORC2 through Rac1, which in turn synergistically increases glucose consumption and glycolysis gene expression (Esen et al., 2013). The deletion of Rictor, which is unique to the mTORC2 signaling pathway, will reduce bone formation in the physiological state and weaken the anabolism of anti-sclerostin therapy, which aims to promote the interaction between Wnt and the receptor (Chen et al., 2015; Sun et al., 2016a). These results indicate the central function of mTORC2 in Wnt-induced osteogenesis and osteoblast metabolism. A recent study found that Wnt3a promoted the phosphorylation of Akt, activating mitochondrial OXPHOS levels in osteoblast lineage cells (ST2 and MC3T3-E1) (Smith and Eliseev, 2021). Wnt7b increases the expression of GLUT1 and the consumption of glucose in primary osteoblast lineage cells, and the loss of GLUT1 reduces the differentiation of osteoblasts *in vitro* (Chen et al., 2019). The non-canonical Wnt5a signaling pathway increases the expression of LRP5/6 to promote osteoblast differentiation. Wnt5a^+/-^ mice have low bone mass and impaired differentiation of osteoblasts and osteoclasts (Maeda et al., 2012). The mechanism by which Wnt7b and Wnt5a recombine in osteoblast metabolism needs further exploration. In the process of osteoblast differentiation, Wnt3a increases aerobic glycolysis and rapidly promotes massive glutamine consumption via activated GLS driven by increasing mTORC1. Isotopic labeling showed that glutamine was metabolized into citrate after entering the TCA cycle. Wnt induces a decrease in glutamine, triggers the GCN2-induced integrated stress response (ISR) pathway as a receptor of amino acid deficiency, and stimulates gene expression related to the transport of amino acid, tRNA aminoacylation, and protein folding. This finding indicates that the catabolism of glutamine not only provides energy for cells but also regulates the process of protein translation as feedback of the intracellular Wnt signal (Frey et al., 2015). Shen found two amino acid transporters: γ (+)—LAT1 and ASCT2 (encoded by slc7 A7 and SLC1A5, respectively), ASCT2 mediates the majority of glutamine uptake, whereas γ (+) - LAT1 in response to Wnt/*ß*- Catenins to promote the expression of SLC7A7 to transport glutamine, whereas the expression of SLC1A5 is regulated by mTORC1/ATF4 (Shen et al., 2021). Frey (Frey et al., 2015; Frey et al., 2018) showed that the fatty acid metabolism in osteoblasts decreases after LRP5 deletion, but the expression of high bone mass variants of LRP5 increases fatty acid metabolism. Only ligands, such as Wnt3a, Wnt10b, and Wnt16, can induce *ß*-catenin to activate fatty acid oxidation, which indicates that Wnt-induced fatty acid metabolism is carried out through the canonical mechanism (Ayturk et al., 2013; Tan et al., 2014).

### The Effect of Hypoxia Inducible Factor on Energy Metabolism

HIFs are regulatory factors that can reduce intracellular oxygen concentration. The expression of HIF can regulate metabolism by regulating the expression of enzymes. Besides hypoxia, some signaling pathways and cytokines can also induce the expression of HIF (Semenza, 2012). In all hMSCs studied, HIF overexpression in a normoxic environment was accompanied by increased glycolysis and HIF target gene expression. This caused a decrease in the expression of HIF-1α and glycolysis mRNA, which led to the weakening of glycolysis and the induction of oxidative metabolism during the osteogenic differentiation of hMSCs. It may also induce mitochondrial biogenesis, a change in mitochondrial morphology and size, an increase in OXPHOS, and extracellular matrix synthesis (Palomäki et al., 2013). Therefore, the osteogenic BMSCs downregulate HIF-1, which is necessary for OXPHOS activation (Karner et al., 2016). Regan et al. (2014) found that the levels of some key enzymes (HK2, 3-phosphoinositide dependent protein kinase-1(PDK1), and LDHA) were upregulated in the bone tissues of mice during glycolysis with the overexpression of HIF-1α in the osteoblast. The activation of HIF-1α increased glycolysis and the high bone mass phenotype. However, the inhibition of PDK1 with DCA did not reverse the high bone mass phenotype in mice. HIF prolyl hydroxylase can hydroxylate the specific residues of HIF protein under oxygen enriched conditions, making it a substrate and proteasome degradation target of E3 ubiquitin ligase von Hippel Lindau (VHL). Osteoblasts lacking VHL showed decreased ubiquitination degradation of HIF, which increased glucose uptake and glycolysis related to target gene expression, high bone mass, hypoglycemia, and increased glucose tolerance. DCA can restore glucose metabolism in VHL-deficient osteoblasts *in vitro* but cannot reverse the high bone mass phenotype (Dirckx et al., 2018). HIF-1 can upregulate glycolytic enzymes, increase glycolytic flux, and attenuate the flux of OXPHOS by reducing the entry of pyruvate into the TCA cycle via the increase in (PDK1) expression (Kierans and Taylor, 2021). Exchange of subunit 4-1 of cytochrome c oxidase (cox4-1) by HIF-1 for the more efficient cox4-2 isoform increases the efficiency of electron flux through the respiratory chain. This increases the efficiency of ATP generation and reduces mitochondria associated ROS generation (Tormos and Chandel, 2010). ROS produced by oxidative stress and apoptosis can be attenuated by targeted binding of HIF-1α to mitochondria to suppress the expression of mRNAs encoded by mitochondrial DNA (Li et al., 2019). HIF-1α stimulates glutathione synthesis mediated by glutaminase, which can neutralize reactive oxygen, maintain a dynamic redox balance at baseline and during oxidative or nutritional stress, increase glycogen storage, prevent energy shortage during a lack of nutrition or oxygen, and ultimately improve the survival rate of transplanted bone cells (Stegen et al., 2016). These studies found that HIF-1α-induced changes in the cell metabolic phenotype are conducive to osteogenic differentiation and promote biosynthesis, but this metabolic change may not be the decisive factor in the high bone mass phenotype. However, a studies have reached mixed conclusions, with the PGE2 receptor subtype 1 (EP1) maintaining high HIF1-α activity to prevent the metabolic shift towards OXPHOS. Deletion of EP1 results in HIF1-α inactivation, increasing the oxygen consumption rate and thus promoting osteogenesis (Feigenson et al., 2017). HIF1-α affecting the differentiation of osteoblast through energy metabolic pathways warrants further investigation.

### The Effects of Other Signaling Pathways on Energy Metabolism

**FIGURE 1:**
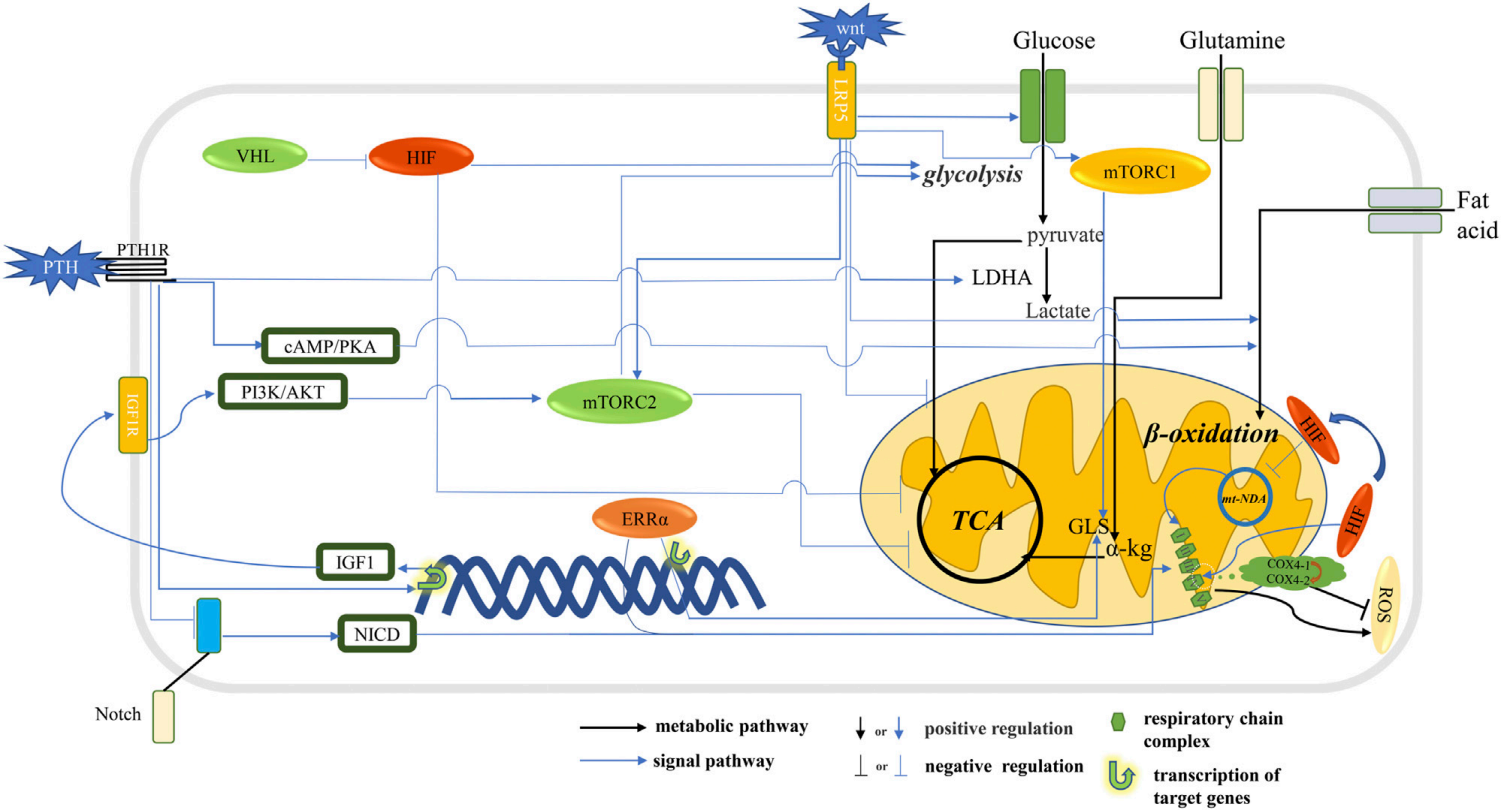
Signal pathway that affects metabolic pathways in osteoblast. The HIF stimulates glycolysis and inhibits oxidative phosphorylation. The Wnt signaling pathway and PTH stimulate glycolysis and inhibit oxidative phosphorylation through mTORC2 and promote the *ß*-oxidation of fatty acids. Notch signaling pathway and ERRα stimulate the expression of respiratory chain complex and promote mitochondrial OXPHOS. The Wnt signaling pathway and ERRα stimulates GLS expression, thereby promoting the conversion of glutamine to α-kg into the TCA cycle. The HIF signaling pathway promotes glycolysis flux and inhibits TCA cycling and reduces ROS production.

Hedgehog (Hh) signaling can regulate bone formation and the osteogenic differentiation of BMSCs and can also quickly induce Warburg effects, such as metabolic reorganization, by activating the cilium-dependent Smo-Ca2^+^-AMPK axis and driving intense insulin independent glucose intake in muscle and brown adipose tissue (Teperino et al., 2012). Although there is no research illustrating the influence of the Hh signal on energy metabolism during osteoblast differentiation, it has been proved that the expression of IGF2 induced by the Hh signal through Gli2 leads to the activation of mTROC2 in osteoblast progenitor cells (Shi et al., 2015). Therefore, it is valuable to investigate whether there is a relationship between Hh signaling and intracellular metabolic remodeling during bone formation and study the specific regulatory mechanism. Conditional deletion of the Notch2 receptor in mesenchymal precursor cells leads an increase in bone mass, which confirms the function of Notch in the bone, and continuous activation of the Notch2 signal in osteoblasts leads to osteopenia in mice (Zanotti et al., 2017). When Notch2 is activated, the transcription complex formed by the Notch intracellular domain reduces the expression of the glycolytic enzyme and mitochondrial respiratory protein. The inhibition of mitochondrial complex 1 can also lead to a reduction in intracellular AMPK activity that further inhibits glycolysis. One of the mechanisms of Notch on bone is to reduce the differentiation of osteoblasts by inhibiting the glycolysis of early osteoblasts. Loss of Notch2 results in high bone mass and enhanced glycolysis. In addition, 3-(3-pyridyl)-1-(4-pyridyl)-2-propene-1-one (3PO) inhibits the reduction in glycolysis by PFKFB3, thus reversing the excessive bone formation induced by a Notch2 deficiency (Lee and Long, 2018). One study showed that PTH inhibits the osteogenic Notch signaling pathway. Therefore, it is meaningful to explore the synergistic effect of two signals during the recombination of metabolism in osteoblasts (Zanotti and Canalis, 2017). Peroxisome proliferator activated receptor δ (PPARδ) is critical for the metabolic adaptation of osteoblasts and the increase in mitochondrial respiration. The specific PPARδ deletion of osteoblasts in mice caused a change in energy homeostasis and impaired mineralization and osteopenia in osteoblasts (Müller et al., 2020). In BMSCs, GOLM1 overexpression stimulates the mTOR signaling pathway, increases glutamate dehydrogenase activity and glutamine conversion to α-kg, and inhibits the osteogenic differentiation of BMSCs (Shen et al., 2018). In addition, BMP indirectly regulates energy metabolism and bone homeostasis through some signaling pathways such as mTOR, HIF, PTH and Wnt. But the effect on metabolic phenotypes needs to be confirmed in more experiments (Yang et al., 2020) (see Figure 1).

## The Effect of the Intermediate Products of Energy Metabolism on Osteogenesis

Cell signaling pathways and energy metabolism are not only independent entities but are also closely linked. Although it is known that energy metabolism is regulated by signal transduction, signal pathways and transcription networks can be regulated by protein post-translational modification, and the intermediate products of energy metabolism are the substrates involved in protein modification. α-kg, which is produced in the TCA cycle of mitochondria, maintains the polymorphism and differentiation of primordial germ cells by retaining specific histone methylation (Tischler et al., 2019). Acetyl CoA provides the acetyl group for histone acetylation modification (Wellen and Thompson, 2012), and glucose can produce UDP-GlcNAc through the hexosamine biosynthesis pathway as a substrate for O-GlcNAc modification (Tan et al., 2018). Although DNA methylation participates in the regulation of gene expression related to osteogenesis (Cheishvili et al., 2018), the regulatory role of DNA methylation at the substrate level is rarely reported. Therefore, we mainly discuss the effects of acetylation and O-GlcNAc modification on osteogenesis at the substrate level. As an important product of energy metabolism, ROS have an important regulatory effect on signaling proteins and transcription factors.

### α-Kg

α-Kg regulates the expression of corresponding signaling molecules by participating in histone methylation. Histone methyltransferases (HMT) participate in the methylation of histones using SAM as a methyl donor. in contrast, histone demethylases remove methyl groups. Demethylases containing Jumonji domain (JHDM) consumed α- KG and O_2_ in demethylation reactions. Some metabolites in the TCA cycle, such as succinate, fumarate are inhibitors of demethylase activity. Furthermore, 2-hydroxyglutarate (2HG), generated from the two electron reduction of α- KG, can also inhibit demethylation, and thus, histone methylation is critically involved in energy metabolism (Su et al., 2016; Ye and Tu, 2018). Studies have shown that exogenous supplementation α Kg can effectively improve bone mass, reduce bone loss, and promote bone regeneration in aging mice. α Kg decreased the abundance of h3k9me3 and H3K27me3, leading to decreased enrichment in the promoter regions of BMP2, BMP4, and Nanog and subsequent upregulation of BMP and Nanog expression (Song et al., 2019). Exogenous α Kg did not significantly promote osteoblast proliferation, but it activated JNK and mTOR/S6K1/S6 signaling pathways to upregulate the expression of osteogenic transcription factors Runx2 and osterix (Żurek et al., 2019).

### Acetylation Modification

Histone acetyltransferase and histone deacetylase can regulate the acetylation of histone lysine residues, which are essential for osteogenic differentiation (Puttagunta et al., 2014; Ortega et al., 2018). Recent studies have revealed that histone modification can determine the direction of the differentiation lineage of MSCs (Huang et al., 2015), such as histone acetylation modification in the promoter regions of Runx2 and OSX and peroxisome proliferator activated receptor γ(PPARγ) and CCAAT enhancer–binding protein α(CEBPA) in osteogenic differentiation. P300/CBP-associated factor (PCAF) is a kind of H3K9 acetyltransferase that is recruited into the promoters of BMP2, BMP3, BMPR1b, and Runx2. It is important to note that the deletion of the PCAF gene will seriously damage the osteogenic differentiation of MSCs. In addition, GCN5, known as lysine acetyltransferase 2 A (KAT2A), is a paragenetic homolog of PCAF that also stimulates bone formation (Gao et al., 2016). Acetyl CoA, which is produced in the TCA cycle of the mitochondria, is the key cofactor of acetylation, can provide the acetyl group for histone acetyltransferase, and is known to exist in the mitochondria and cytoplasm. Once acetyl CoA produced by the mitochondria enters the TCA cycle, it will be converted into citrate that can leave the mitochondria. Intracellular citric acid is converted to acetyl CoA by ATP citrate lyase (ACL) (Wellen and Thompson, 2012). Karner et al. (2015) showed that the decrease in nuclear acetyl CoA can inhibit the gene expression of osteoblasts. This may be one of the mechanisms by which cells need to transfer into glycolysis during osteogenic differentiation. Therefore, controlling metabolic pathways and increasing or decreasing the flux of these substrates controls energy production and gene expression via epigenetics. The availability of these energy-producing substances and the ATP demand of cells are compatible with the signaling pathways that need to be upregulated or downregulated.

The level of histone acetylation of the Wnt genes (Wnt1, Wnt6, Wnt10a, and Wnt10b) decreased in the BMSCs of ovariectomized mice. It was found that the expression of histone acetyltransferase was upregulated when BMSCs differentiated into osteoblasts but downregulated after induced osteoporosis. This is because GCN5 increases the acetylation of the histone 3-lysine 9 site on the Wnt gene promoter to induce osteogenic differentiation of BMSCs. The decrease in GCN5 expression inhibits Wnt signal transduction, leading to the weakening of the osteogenesis of BMSCs in ovariectomized mice (Jing et al., 2018). GCN5 also promoted BMSC-mediated angiogenesis by increasing the level of H3K9Ac on the vascular endothelial growth factor 5(VEGF5) promoter. Overexpression of GCN5 enhanced the angiogenetic ability of osteoporotic BMSCs (Jing et al., 2017). The metabolic changes regulated by Wnt may also affect the differentiation and function of osteoblasts through epigenetic modification. After ST2 cells were stimulated with Wnt3a, more genes were downregulated, such as PPARγ and CEBPA, which encode adipocyte specific transcription factors and prevent them from inducing osteoblast differentiation, causing a decrease in the level of histone acetylation, which is related to decreased levels of nuclear acetyl CoA (histone acetyltransferase substrate) and citrate (precursor of acetyl CoA synthesis) because the Wnt signaling pathway increases aerobic hydrolysis and upregulates the expression of PDK1, thus inhibiting pyruvate from entering the TCA and decreasing the availability of nuclear acetyl CoA (Karner et al., 2016). The above studies showed that the increase in the acetylation level of the histones of specific osteogenic genes can promote osteogenic differentiation, but for BMSCs in which OXPHOS is not very active, it is beneficial to reduce acetylation through aerobic fermentation because it also inhibits the expression of non-osteogenic genes, which are related to adipogenic and chondrogenic differentiation and indirectly induce osteoblast differentiation. However, Shares et al. (2018) found that the osteogenic ability of BMSCs treated with an OXPHOS inhibitor decreased, but the ATP level did not decrease because of the glycolysis compensation mechanism. In addition, it was found that active mitochondria promote the differentiation of osteoblasts by promoting the acetylation of *ß*-catenin. From these studies, we found that BMSCs can regulate the acetylation level of protein through different energy metabolism channels in the early and late stages of osteogenic differentiation, and then osteogenic differentiation is programmed synchronously.

### O-GlcNAc Modification

O-GlcNAc can be regulated as a nutritional response through hexosamine biosynthesis. UDP-GlcNAc produced by this pathway is the substrate of O-GlcNAc modification, which is catalyzed reversibly by O-GlcNAc transferase (OGT) and O-linked n-acetylglucosaminidase (OGA). It is worth noting that OGA deletion is lethal to perinatal mice (Keembiyehetty et al., 2015). Approximately 3% glucose enters the hexosamine pathway for various biochemical reactions to regulate cell performance, which may be sensitive to the content of some nutrients, such as glucose, glutamine, and acetyl CoA. Therefore, the UDP-GlcNAc pool is reduced and O-GlcNAc modification is affected when glucose uptake is limited (Wellen and Thompson, 2012). O-GlcNAc modification is an inducible, reversible, and dynamic post-translational modification of protein. The main modification sites are located on the serine and threonine residues of the target protein. O-GlcNAc modifies almost every functional class of nuclear and cytoplasmic proteins, which includes the complex processes of cell transcription, translation, signal transduction, and material metabolism. Abnormal O-GlcNAc modification is related to the progression of diseases, such as cancer, neurodegeneration, and diabetes (Banerjee et al., 2016). In addition, the level of O-GlcNAc modification must be maintained within an optimal range to maintain normal cell function (Yang and Qian, 2017).

O-GlcNAc affects lineage distribution during cell differentiation. One study showed that the total intracellular O-GlcNAc level changes during the adipogenic, chondrogenic, and osteogenic differentiation of BMSCs (Sun et al., 2016b). Kim et al. (2007) found that O-GlcNAc upregulates the expression of osteocalcin by modifying the transcriptional activity of Runx2 and OSE2 during osteoblast differentiation. Protein-related O-GlcNAc increased in MC3T3-E1 cells during osteogenic differentiation. The expression of osteocalcin induced by ascorbic acid, PTH, and diphtheria can be enhanced by PUGNAc, an OGA inhibitor. The OSE2 and Runx2 sites in the osteocalcin promoter are important factors for PUGNAc to enhance the osteocalcin promoter activity, and PUGNAc can also increase the O-GlcNAc modification of Runx2; therefore, osteocalcin is its target gene in which transcription will be regulated. Nagel and Ball (2014) conducted further research that characterized Runx2 via electron transfer dissociation tandem mass spectrometry and revealed the site of O-GlcNAc modification, which is a post-translational modification of the nutritional response, regulating the function of transcription effectors. O-GlcNAc modification occurs near the known sites regulating phosphorylation residues and arginine methylation in the transactivated region of Runx2. Runx2 was also detected in the interaction between OGT and O-GlcNAc. The inhibition of OGA (responsible for scavenging O-GlcNAc in Ser/Thr residues) can enhance the basic transcriptional activity of MC3T3-E1 osteoblasts and the transcriptional activity of Runx2 induced by BMP2/7, resulting an increase in ALP expression and activity. Koyama and Kamemura (2015) also found that the expression of ALP, OCN, and bone sialoprotein (BSP) was inhibited or promoted by OGA and OGT, respectively during the osteogenic differentiation of MC3T3-E1 cells. But Gu et al. (2018) found that after C2C12 cells were treated with high glucose, glucosamine, or N-acetylglucosamine, the O-GlcNAc level and total protein level of Runx2 increased, which led to a decrease in Runx2 transcription activity and the expression level of osteogenic marker genes. Therefore, we know that the expression of osteoblast-related genes will be reduced if O-GlcNAc modification is insufficient or excessive, and appropriate O-GlcNAc modification is more conducive to osteogenesis. In the future, we can further explore the appropriate abundance of UDP-GlcNAc, OGA, and OGT in cells in order to maximize the role of osteogenesis differentiation.

### ROS

**FIGURE 2:**
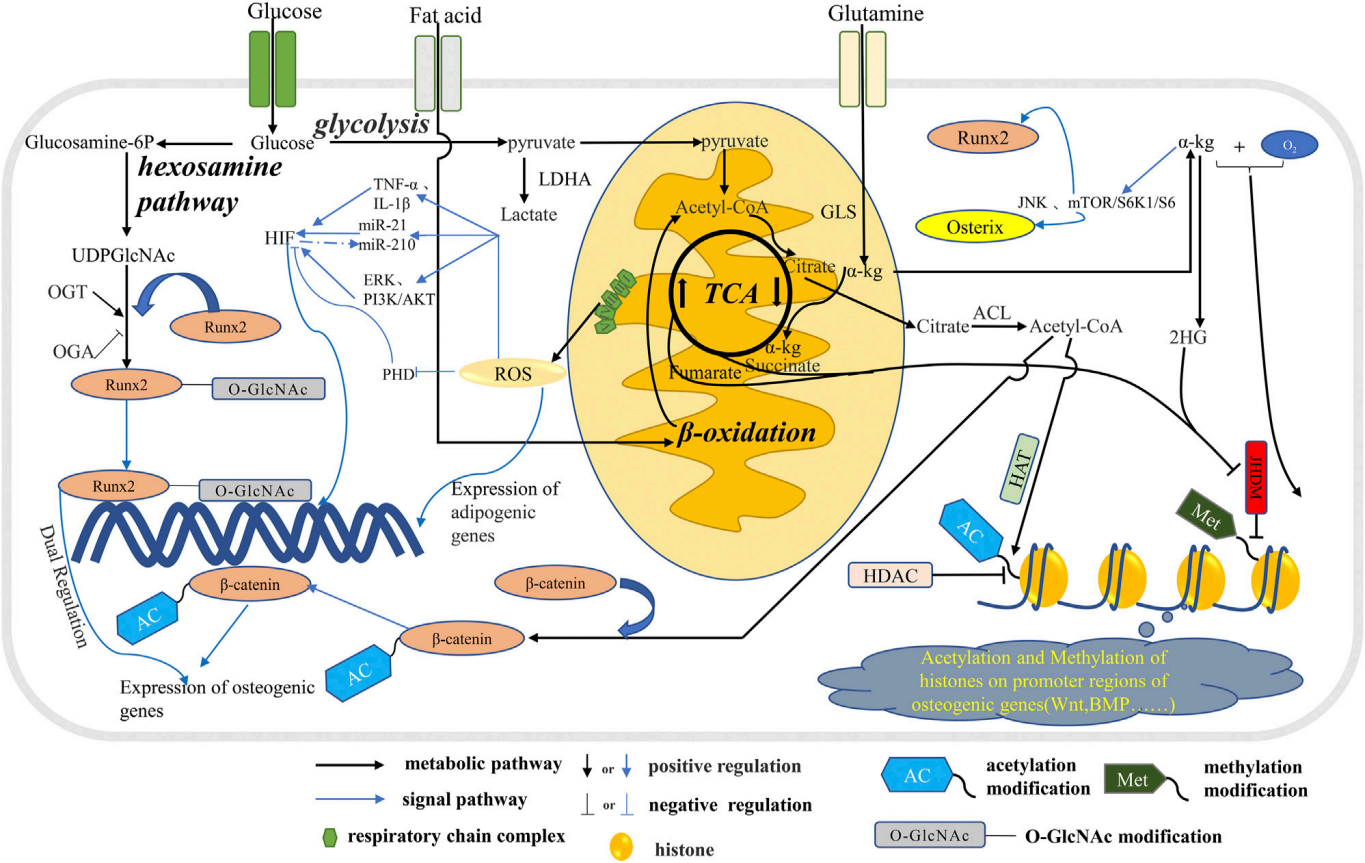
The metabolic pathways in osteoblast affect signal transduction. Glucose, fatty acids and glutamine are metabolized through the tricarboxylic acid cycle in mitochondria to produce citric acid, which is metabolized into acetyl-CoA in the cytoplasm. Part of acetyl-CoA enters the nucleus to participate in the acetylation of histones and regulate the expression of related genes. A portion of acetyl-CoA participate the *ß*-catenin acetylation which regulate the expression of osteogenic genes. Glucose produces UDPGlcNAc through the hexosamine pathway, which participates in the O-GlcNA modification of osteogenic transcription factor Runx2 and regulates the expression of osteogenic genes. In addition, ROS produced during mitochondrial OXPHOS inhibits osteogenesis by stimulating the expression of adipogenic genes. In addition, ROS further influences the energy metabolism phenotype in osteoblasts by regulating HIF through multiple pathways. α-Kg produced by glutamine and TCA cycles promotes osteogenic differentiation by means of histone methylation and JNK and mTOR/S6K1/S6 pathways.

OXPHOS and OCR increase and mitochondrial function is enhanced during osteogenic differentiation. The mitochondrial respiratory chain complex is the main production site of ROS, particularly complexes I and III. In addition, ROS is produced in various metabolic reactions involving PDH and electron transfer flavin (ETF) (Holzerová and Prokisch, 2015). In the process of osteogenic differentiation, ROS is produced due to the large demand for energy, and it is a crucial factor in regulating the osteogenic differentiation of BMSCs (Geng et al., 2019). The older the BMSCs, the more free ROS will destroy the distribution of lineage, which will promote fat formation and hinder bone formation (Callaway and Jiang, 2015). Osteogenic and adipogenic differentiation are inhibited by increased ROS induced by the lack of superoxide dismutase 2 in stromal precursor cells. The toxic accumulation of α-ketoglutarate in cells leads to nuclear plasma vacuolation and chromatin concentration, which significantly inhibits osteogenesis and adipocyte differentiation. At the same time, the accompanying DNA damage, the increase in HIF-1 degradation, and the inhibition of acetylated histone H3 (Lys27) all lead to the death of BMSCs (Singh et al., 2017). When BMSCs have just differentiated into adipogenic cells, an increase in intracellular ROS and the overexpression of PPAR γ were observed, and this is necessary to start adipocyte differentiation. The formation of adipocytes induced by arabinosylcytosine is related to the increase in ROS level in the cytoplasm because antioxidant N-acetyl-l-cysteine (NAC) can reduce adipocyte production by scavenging intracellular ROS. However, NAC has no obvious blocking effect on ROS and adipogenesis under physiological conditions. Therefore, antioxidants targeting the mitochondria can reduce adipocyte differentiation induced by ROS (Wang et al., 2015). It has been reported that ROS promotes the adipogenic differentiation of rat osteoblasts induced by high glucose via PI3K/Akt signaling (Zhang and Yang, 2013). In addition, uncarboxylated osteocalcin can inhibit ROS production induced by high glucose levels by inhibiting PI3K/Akt signaling in the MC3T3-E1 cells and can stimulate osteoblast differentiation (Liu and Yang, 2016). It is worth noting that there is a complex cross-talk relationship between ROS and HIF-1a signaling. Movafagh scrutinized the regulation of HIF-1a by ROS. ROS can inhibit the activity of prolyl hydroxylase PHD to stabilize HIF-1a. HIF-1a expression by ROS is mediated through the ERK and PI3K/Akt pathways, and ROS increase the inflammatory mediator TNF-α and IL-1β And Mir-21 to activate the ERK and PI3K/Akt pathways to upregulate HIF-1a, in addition, Mir-210 has a complex feedback regulatory mechanism on ROS and HIF-1a (Movafagh et al., 2015) (see Figure 2).

## Conclusion

Understanding the mechanism of osteoblast differentiation and function is the key to discovering a new bone synthesis therapy method. For example, a recent study revealed that the use of oxalate, an LDH inhibitor, can convert the biological energy of bone progenitor cells from glycolysis to oxidative phosphorylation *in vitro* and increase their osteogenic differentiation potential. *In vitro* studies have also proved that oxalate can increase bone mineral density, improve cortical bone structure, and enhance bone strength (Hollenberg et al., 2020). Sodium butyrate (NaB) can upregulate PGC-1 α And TFAM by Nrf2/GSK-3 *ß* Signaling to prevented H2O2 induced oxidative damage in MC3T3-E1 cells, which can promote osteoblast mineralization and differentiation, and maintained the balance of bone metabolism by increasing cellular antioxidant capacity, ATP production, and reducing ROS levels (Tang et al., 2020). It is also worth further sturdy in the field of bone regeneration to investigate the effects of the regenerated biomaterials on energy metabolism (Fan et al., 2014; Liao et al., 2017; Marew and Birhanu, 2021; Graeff et al., 2022).

According to the previous discussion, the effects of the signaling pathways on osteoblast energy metabolism mainly focus on Wnt, PTH, and ERRα, and the effects of other signaling pathways need to be further explored. mTOR is the central target related to metabolism in the process of osteoblast differentiation, and IGF1 and HIF-1α are also significant regulatory factors. Current research primarily focuses on the metabolism of glucose. Glucose produces ATP through glycolysis and OXPHOS, while catabolic products provide a carbon source for other anabolic metabolism and substrates for protein modification to regulate its function and gene expression. The direction of glucose metabolism (producing energy or intermediate metabolites) seems to control the direction of cell differentiation and its final outcome, but this needs further confirmation. It is necessary to further study whether other signaling pathways exist, in addition to the Wnt signaling pathway, that can change the level of acetyl CoA through intracellular glucose metabolism to regulate the acetylation of histones and indirectly regulate the expression of related genes. The process of osteoblast differentiation and lineage distribution during the differentiation of BMSCs causes continuous reorganization of the energy metabolism pathways, which leads to changes in intracellular nutritional status. In addition, O-GlcNAc modification is very sensitive to the availability of nutrients and the stress response, and the way in which this process stimulates intracellular O-GlcNAc modification has not yet been explored. Another avenue for future research is investigating whether a change in acetyl CoA or UDP-GlcNAc levels can modify some signal proteins to regulate gene expression. One study showed that the nicotinamide adenine dinucleotide (NAD^+^)/silent information regulator 1(SIRT1) axis can regulate the osteogenic differentiation of MSC (Pi et al., 2019), and experiments *in vivo* and vitro have proved that nicotinamide mononucleotide (NAD^+^ precursor) can promote osteogenesis through the SIRT1 pathway (Song et al., 2019). Therefore, it can be speculated that intracellular metabolic recombination causes a change in NAD^+^/reduced form of nicotinamide-adenine dinucleotide (NADH), which leads to osteogenic differentiation through the SIRT1 pathway, but further proof is needed. The energy metabolism of fatty acids and amino acids needs further study. The bioenergetic characteristics of various signal transduction mechanisms and the stages of osteoblast differentiation are different. Therefore, the relationship between energy metabolism and bone formation is extremely complex.

For the foreseeable future, the application of multiomics technologies, such as transcriptomics, proteomics, and metabonomics, can systematically study the interaction between intracellular signaling pathways and metabolic pathways in osteogenesis from different dimensions. The development of corresponding targeted drugs against targets of energy metabolism during osteogenesis to promote bone regeneration is also worth investigating, such as the application of tFNA, and there are current studies that target mir-2861 to BMSCs to promote osteogenesis by inhibiting the expression of histone deacetylase 5 (hdac5) to maintain histone acetylation (Li et al., 2021; Zhang et al., 2021). The in-depth exploration of the interaction between intracellular energy metabolism and signaling pathways during osteogenesis will promote the development of basic biology and provide new therapeutic targets and strategies for osteoporosis.

## References

[B1] Adamek G. Felix R. Guenther H. L. Fleisch H. (1987). Fatty Acid Oxidation in Bone Tissue and Bone Cells in Culture. Characterization and Hormonal Influences. Biochem. J. 248 (1), 129–137. 10.1042/bj2480129 3325035 PMC1148509

[B2] Ayturk U. M. Jacobsen C. M. Christodoulou D. C. Gorham J. Seidman J. G. Seidman C. E. (2013). An RNA‐seq Protocol to Identify mRNA Expression Changes in Mouse Diaphyseal Bone: Applications in Mice with Bone Property Altering Lrp5 Mutations. J. Bone Miner Res. 28 (10), 2081–2093. 10.1002/jbmr.1946 23553928 PMC3743099

[B3] Balani D. H. Ono N. Kronenberg H. M. (2017). Parathyroid Hormone Regulates Fates of Murine Osteoblast Precursors *In Vivo* . J. Clin. Invest. 127 (9), 3327–3338. 10.1172/jci91699 28758904 PMC5669555

[B4] Banerjee P. S. Lagerlöf O. Hart G. W. (2016). Roles of O-GlcNAc in Chronic Diseases of Aging. Mol. Aspects Med. 51, 1–15. 10.1016/j.mam.2016.05.005 27259471

[B5] Bonnelye E. Aubin J. E. (2013). An Energetic Orphan in an Endocrine Tissue: a Revised Perspective of the Function of Estrogen Receptor-Related Receptor Alpha in Bone and Cartilage. J. Bone Miner Res. 28 (2), 225–233. 10.1002/jbmr.1836 23212690

[B6] Bott A. Maimouni S. Zong W.-X. (2019). The Pleiotropic Effects of Glutamine Metabolism in Cancer. Cancers 11 (6), 770. 10.3390/cancers11060770 PMC6627534 31167399

[B7] Callaway D. A. Jiang J. X. (2015). Reactive Oxygen Species and Oxidative Stress in Osteoclastogenesis, Skeletal Aging and Bone Diseases. J. Bone Miner Metab. 33 (4), 359–370. 10.1007/s00774-015-0656-4 25804315

[B8] Campbell S. E. Febbraio M. A. (2001). Effect of Ovarian Hormones on Mitochondrial Enzyme Activity in the Fat Oxidation Pathway of Skeletal Muscle. Am. J. Physiology-Endocrinology Metab. 281 (4), E803–E808. 10.1152/ajpendo.2001.281.4.e803 11551858

[B9] Cheishvili D. Parashar S. Mahmood N. Arakelian A. Kremer R. Goltzman D. (2018). Identification of an Epigenetic Signature of Osteoporosis in Blood DNA of Postmenopausal Women. J. Bone Miner Res. 33 (11), 1980–1989. 10.1002/jbmr.3527 29924424

[B10] Chen H. Ji X. Lee W.-C. Shi Y. Li B. Abel D. (2019). Increased Glycolysis Mediates Wnt7b‐induced Bone Formation. FASEB j. 33 (7), 7810–7821. 10.1096/fj.201900201rr 30913395 PMC6593878

[B11] Chen J. Holguin N. Shi Y. Silva M. J. Long F. (2015). mTORC2 Signaling Promotes Skeletal Growth and Bone Formation in Mice. J. Bone Miner Res. 30 (2), 369–378. 10.1002/jbmr.2348 25196701 PMC4322759

[B12] DeBerardinis R. J. Mancuso A. Daikhin E. Nissim I. Yudkoff M. Wehrli S. (2007). Beyond Aerobic Glycolysis: Transformed Cells Can Engage in Glutamine Metabolism that Exceeds the Requirement for Protein and Nucleotide Synthesis. Proc. Natl. Acad. Sci. 104 (49), 19345–19350. 10.1073/pnas.0709747104 18032601 PMC2148292

[B13] Dirckx N. Moorer M. C. Clemens T. L. Riddle R. C. (2019). The Role of Osteoblasts in Energy Homeostasis. Nat. Rev. Endocrinol. 15 (11), 651–665. 10.1038/s41574-019-0246-y 31462768 PMC6958555

[B14] Dirckx N. Tower R. J. Mercken E. M. Vangoitsenhoven R. Moreau-Triby C. Breugelmans T. (2018). Vhl Deletion in Osteoblasts Boosts Cellular Glycolysis and Improves Global Glucose Metabolism. J. Clin. Invest. 128 (3), 1087–1105. 10.1172/jci97794 29431735 PMC5824856

[B15] Esen E. Chen J. Karner C. M. Okunade A. L. Patterson B. W. Long F. (2013). WNT-LRP5 Signaling Induces Warburg Effect through mTORC2 Activation during Osteoblast Differentiation. Cell Metab. 17 (5), 745–755. 10.1016/j.cmet.2013.03.017 23623748 PMC3653292

[B16] Esen E. Lee S.-Y. Wice B. M. Long F. (2015). PTH Promotes Bone Anabolism by Stimulating Aerobic Glycolysis via IGF Signaling. J. Bone Miner Res. 30 (11), 1959–1968. 10.1002/jbmr.2556 25990470 PMC4825329

[B17] Fan J. Kamphorst J. J. Mathew R. Chung M. K. White E. Shlomi T. (2013). Glutamine‐driven Oxidative Phosphorylation Is a Major ATP Source in Transformed Mammalian Cells in Both Normoxia and Hypoxia. Mol. Syst. Biol. 9, 712. 10.1038/msb.2013.65 24301801 PMC3882799

[B18] Fan L. Li J. Yu Z. Dang X. Wang K. (2014). The Hypoxia-Inducible Factor Pathway, Prolyl Hydroxylase Domain Protein Inhibitors, and Their Roles in Bone Repair and Regeneration. Biomed. Res. Int. 2014, 239356. 10.1155/2014/239356 24895555 PMC4034436

[B19] Fan Y. Hanai J.-i. Le P. T. Bi R. Maridas D. DeMambro V. (2017). Parathyroid Hormone Directs Bone Marrow Mesenchymal Cell Fate. Cell Metab. 25 (3), 661–672. 10.1016/j.cmet.2017.01.001 28162969 PMC5342925

[B20] Feigenson M. Eliseev R. A. Jonason J. H. Mills B. N. O'Keefe R. J. (2017). PGE2 Receptor Subtype 1 (EP1) Regulates Mesenchymal Stromal Cell Osteogenic Differentiation by Modulating Cellular Energy Metabolism. J. Cell. Biochem. 118 (12), 4383–4393. 10.1002/jcb.26092 28444901 PMC5774856

[B21] Frey J. L. Kim S. P. Li Z. Wolfgang M. J. Riddle R. C. (2018). β-Catenin Directs Long-Chain Fatty Acid Catabolism in the Osteoblasts of Male Mice. Endocrinology 159 (1), 272–284. 10.1210/en.2017-00850 29077850 PMC5761587

[B22] Frey J. L. Li Z. Ellis J. M. Zhang Q. Farber C. R. Aja S. (2015). Wnt-Lrp5 Signaling Regulates Fatty Acid Metabolism in the Osteoblast. Mol. Cell Biol 35 (11), 1979–1991. 10.1128/mcb.01343-14 25802278 PMC4420919

[B23] Gallet M. Saïdi S. Haÿ E. Photsavang J. Marty C. Sailland J. (2013). Repression of Osteoblast Maturation by ERRα Accounts for Bone Loss Induced by Estrogen Deficiency. PLoS One 8 (1), e54837. 10.1371/journal.pone.0054837 23359549 PMC3554601

[B24] Gao F. Chiu S. M. Motan D. A. L. Zhang Z. Chen L. Ji H.-L. (2016). Mesenchymal Stem Cells and Immunomodulation: Current Status and Future Prospects. Cell Death Dis 7–e2062. 10.1038/cddis.2015.327 PMC4816164 26794657

[B25] Geng W. Shi H. Zhang X. Tan W. Cao Y. Mei R. (2019). Substance P Enhances BMSC Osteogenic Differentiation via Autophagic Activation. Mol. Med. Rep. 20 (1), 664–670. 10.3892/mmr.2019.10257 31115537 PMC6580032

[B26] Gerhart-Hines Z. Dominy J. E. Jr Blättler S. M. Jedrychowski M. P. Banks A. S. Lim J.-H. (2011). The cAMP/PKA Pathway Rapidly Activates SIRT1 to Promote Fatty Acid Oxidation Independently of Changes in NAD+. Mol. Cell 44 (6), 851–863. 10.1016/j.molcel.2011.12.005 22195961 PMC3331675

[B27] Graeff M. S. Z. Tokuhara C. K. Sanches M. L. R. Buzalaf M. A. R. Rocha L. A. Oliveira R. C. (2022). Osteoblastic Response to Biomaterials Surfaces: Extracellular Matrix Proteomic Analysis. J. Biomed. Mater. Res. 110 (1), 176–184. 10.1002/jbm.b.34900 34196101

[B28] Gu H. Song M. Boonanantanasarn K. Baek K. Woo K. Ryoo H.-M. (2018). Conditions Inducing Excessive O-GlcNAcylation Inhibit BMP2-Induced Osteogenic Differentiation of C2C12 Cells. Ijms 19 (1), 202. 10.3390/ijms19010202 PMC5796151 29315243

[B29] Guntur A. R. Le P. T. Farber C. R. Rosen C. J. (2014). Bioenergetics during Calvarial Osteoblast Differentiation Reflect Strain Differences in Bone Mass. Endocrinology 155 (5), 1589–1595. 10.1210/en.2013-1974 24437492 PMC3990840

[B30] Hollenberg A. M. Smith C. O. Shum L. C. Awad H. Eliseev R. A. (2020). Lactate Dehydrogenase Inhibition with Oxamate Exerts Bone Anabolic Effect. J. Bone Miner Res. 35 (12), 2432–2443. 10.1002/jbmr.4142 32729639 PMC7736558

[B31] Holzerová E. Prokisch H. (2015). Mitochondria: Much Ado about Nothing? How Dangerous Is Reactive Oxygen Species Production? Int. J. Biochem. Cell Biol 63, 16–20. 10.1016/j.biocel.2015.01.021 25666559 PMC4444525

[B32] Hu G. Yu Y. Tang Y. J. Wu C. Long F. Karner C. M. (2020). The Amino Acid Sensor Eif2ak4/GCN2 Is Required for Proliferation of Osteoblast Progenitors in Mice. J. Bone Miner Res. 35 (10), 2004–2014. 10.1002/jbmr.4091 32453500 PMC7688563

[B33] Huang B. Li G. Jiang X. H. (2015). Fate Determination in Mesenchymal Stem Cells: a Perspective from Histone-Modifying Enzymes. Stem Cell Res Ther 6 (1), 35. 10.1186/s13287-015-0018-0 25890062 PMC4365520

[B34] Huang T. Liu R. Fu X. Yao D. Yang M. Liu Q. (2017). Aging Reduces an ERRalpha‐Directed Mitochondrial Glutaminase Expression Suppressing Glutamine Anaplerosis and Osteogenic Differentiation of Mesenchymal Stem Cells. Stem Cells 35 (2), 411–424. 10.1002/stem.2470 27501743

[B35] Jing H. Liao L. Su X. Shuai Y. Zhang X. Deng Z. (2017). Declining Histone Acetyltransferase GCN5 Represses BMSC‐mediated Angiogenesis during Osteoporosis. FASEB j. 31 (10), 4422–4433. 10.1096/fj.201700118r 28642327

[B36] Jing H. Su X. Gao B. Shuai Y. Chen J. Deng Z. (2018). Epigenetic Inhibition of Wnt Pathway Suppresses Osteogenic Differentiation of BMSCs during Osteoporosis. Cell Death Dis 9 (2), 176. 10.1038/s41419-017-0231-0 29416009 PMC5833865

[B37] Karner C. M. Esen E. Chen J. Hsu F.-F. Turk J. Long F. (2016). Wnt Protein Signaling Reduces Nuclear Acetyl-Coa Levels to Suppress Gene Expression during Osteoblast Differentiation. J. Biol. Chem. 291, 13028–13039. 10.1074/jbc.m115.708578 27129247 PMC4933220

[B38] Karner C. M. Esen E. Okunade A. L. Patterson B. W. Long F. (2015). Increased Glutamine Catabolism Mediates Bone Anabolism in Response to WNT Signaling. J. Clin. Invest. 125 (2), 551–562. 10.1172/jci78470 25562323 PMC4319407

[B39] Karner C. M. Long F. (2017). Wnt Signaling and Cellular Metabolism in Osteoblasts. Cell. Mol. Life Sci. 74 (9), 1649–1657. 10.1007/s00018-016-2425-5 27888287 PMC5380548

[B40] Keembiyehetty C. Love D. C. Harwood K. R. Gavrilova O. Comly M. E. Hanover J. A. (2015). Conditional Knock-Out Reveals a Requirement for O-Linked N-Acetylglucosaminase (O-GlcNAcase) in Metabolic Homeostasis. J. Biol. Chem. 290 (11), 7097–7113. 10.1074/jbc.m114.617779 25596529 PMC4358131

[B41] Kierans S. J. Taylor C. T. (2021). Regulation of Glycolysis by the Hypoxia‐inducible Factor (HIF): Implications for Cellular Physiology. J. Physiol. 599 (1), 23–37. 10.1113/jp280572 33006160

[B42] Kim S.-H. Kim Y.-H. Song M. An S. H. Byun H.-Y. Heo K. (2007). O-GlcNAc Modification Modulates the Expression of Osteocalcin via OSE2 and Runx2. Biochem. Biophysical Res. Commun. 362 (2), 325–329. 10.1016/j.bbrc.2007.07.149 17707335

[B43] Kim S. P. Li Z. Zoch M. L. Frey J. L. Bowman C. E. Kushwaha P. (2017). Fatty Acid Oxidation by the Osteoblast Is Required for normal Bone Acquisition in a Sex- and Diet-dependent Manner. JCI Insight 2 (16), e92704. 10.1172/jci.insight.92704 PMC5621897 28814665

[B44] Koyama T. Kamemura K. (2015). Global Increase in O-Linked N-Acetylglucosamine Modification Promotes Osteoblast Differentiation. Exp. Cell Res. 338 (2), 194–202. 10.1016/j.yexcr.2015.08.009 26302267

[B45] Larsson S. Jones H. A. Göransson O. Degerman E. Holm C. (2016). Parathyroid Hormone Induces Adipocyte Lipolysis via PKA-Mediated Phosphorylation of Hormone-Sensitive Lipase. Cell Signal. 28 (3), 204–213. 10.1016/j.cellsig.2015.12.012 26724218

[B46] Lee S.-Y. Long F. (2018). Notch Signaling Suppresses Glucose Metabolism in Mesenchymal Progenitors to Restrict Osteoblast Differentiation. J. Clin. Invest. 128 (12), 5573–5586. 10.1172/jci96221 30284985 PMC6264656

[B47] Lee W.-C. Guntur A. R. Long F. Rosen C. J. (2017). Energy Metabolism of the Osteoblast: Implications for Osteoporosis. Endocr. Rev. 38 (3), 255–266. 10.1210/er.2017-00064 28472361 PMC5460680

[B48] Li H.-S. Zhou Y.-N. Li L. Li S.-F. Long D. Chen X.-L. (2019). HIF-1α Protects against Oxidative Stress by Directly Targeting Mitochondria. Redox Biol. 25, 101109. 10.1016/j.redox.2019.101109 30686776 PMC6859547

[B49] Li L. Liang Y. Kang L. Liu Y. Gao S. Chen S. (2018). Transcriptional Regulation of the Warburg Effect in Cancer by SIX1. Cancer Cell 33 (3), 368–385. 10.1016/j.ccell.2018.01.010 29455928

[B50] Li S. Liu Y. Tian T. Zhang T. Lin S. Zhou M. (2021). Bioswitchable Delivery of microRNA by Framework Nucleic Acids: Application to Bone Regeneration. Small 17 (47), e2104359. 10.1002/smll.202104359 34716653

[B51] Liao J. Tian T. Shi S. Xie X. Ma Q. Li G. (2017). The Fabrication of Biomimetic Biphasic CAN-PAC Hydrogel with a Seamless Interfacial Layer Applied in Osteochondral Defect Repair. Bone Res. 5, 17018. 10.1038/boneres.2017.18 28698817 PMC5496380

[B52] Lin P.-I. Tai Y.-T. Chan W. P. Lin Y.-L. Liao M.-H. Chen R.-M. (2017). Estrogen/Erα Signaling axis Participates in Osteoblast Maturation via Upregulating Chromosomal and Mitochondrial Complex Gene Expressions. Oncotarget 9 (1), 1169–1186. 10.18632/oncotarget.23453 29416685 PMC5787428

[B53] Liu J. Yang J. (2016). Uncarboxylated Osteocalcin Inhibits High Glucose-Induced ROS Production and Stimulates Osteoblastic Differentiation by Preventing the Activation of PI3K/Akt in MC3T3-E1 Cells. Int. J. Mol. Med. 37 (1), 173–181. 10.3892/ijmm.2015.2412 26719856

[B54] Long F. (2012). Building strong Bones: Molecular Regulation of the Osteoblast Lineage. Nat. Rev. Mol. Cell Biol 13 (1), 27–38. 10.1038/nrm3254 22189423

[B55] Luo C. Balsa E. Thomas A. Hatting M. Jedrychowski M. Gygi S. P. (2017). ERRα Maintains Mitochondrial Oxidative Metabolism and Constitutes an Actionable Target in PGC1α-Elevated Melanomas. Mol. Cancer Res. 15 (10), 1366–1375. 10.1158/1541-7786.mcr-17-0143 28596418 PMC5954239

[B56] Maeda K. Kobayashi Y. Udagawa N. Uehara S. Ishihara A. Mizoguchi T. (2012). Wnt5a-Ror2 Signaling between Osteoblast-Lineage Cells and Osteoclast Precursors Enhances Osteoclastogenesis. Nat. Med. 18 (3), 405–412. 10.1038/nm.2653 22344299

[B57] Marew T. Birhanu G. (2021). Three Dimensional Printed Nanostructure Biomaterials for Bone Tissue Engineering. Regenerative Ther. 18, 102–111. 10.1016/j.reth.2021.05.001 PMC8178073 34141834

[B58] Maridas D. E. Rendina-Ruedy E. Helderman R. C. DeMambro V. E. Brooks D. Guntur A. R. (2019). Progenitor Recruitment and Adipogenic Lipolysis Contribute to the Anabolic Actions of Parathyroid Hormone on the Skeleton. FASEB j. 33 (2), 2885–2898. 10.1096/fj.201800948rr 30354669 PMC6338651

[B59] Meyer J. Salamon A. Mispagel S. Kamp G. Peters K. (2018). Energy Metabolic Capacities of Human Adipose-Derived Mesenchymal Stromal Cells *In Vitro* and Their Adaptations in Osteogenic and Adipogenic Differentiation. Exp. Cell Res. 370 (2), 632–642. 10.1016/j.yexcr.2018.07.028 30036541

[B60] Misra B. B. Jayapalan S. Richards A. K. Helderman R. C. M. Rendina-Ruedy E. (2021). Untargeted Metabolomics in Primary Murine Bone Marrow Stromal Cells Reveals Distinct Profile throughout Osteoblast Differentiation. Metabolomics 17 (10), 86. 10.1007/s11306-021-01829-9 34537901 PMC8450216

[B61] Movafagh S. Crook S. Vo K. (2015). Regulation of Hypoxia-Inducible Factor-1a by Reactive Oxygen Species : New Developments in an Old Debate. J. Cell. Biochem. 116 (5), 696–703. 10.1002/jcb.25074 25546605

[B62] Müller D. I. H. Stoll C. Palumbo-Zerr K. Böhm C. Krishnacoumar B. Ipseiz N. (2020). Pparδ-mediated Mitochondrial Rewiring of Osteoblasts Determines Bone Mass. Sci. Rep. 10 (1), 8428. 10.1038/s41598-020-65305-5 32439961 PMC7242479

[B63] Mulukutla B. C. Yongky A. Le T. Mashek D. G. Hu W.-S. (2016). Regulation of Glucose Metabolism - A Perspective from Cell Bioprocessing. Trends Biotechnol. 34 (8), 638–651. 10.1016/j.tibtech.2016.04.012 27265890

[B64] Nagel A. K. Ball L. E. (2014). O-GlcNAc Modification of the Runt-Related Transcription Factor 2 (Runx2) Links Osteogenesis and Nutrient Metabolism in Bone Marrow Mesenchymal Stem Cells. Mol. Cell Proteomics 13 (12), 3381–3395. 10.1074/mcp.m114.040691 25187572 PMC4256491

[B65] Ortega E. Rengachari S. Ibrahim Z. Hoghoughi N. Gaucher J. Holehouse A. S. (2018). Transcription Factor Dimerization Activates the P300 Acetyltransferase. Nature 562 (7728), 538–544. 10.1038/s41586-018-0621-1 30323286 PMC6914384

[B66] Palomäki S. Pietilä M. Laitinen S. JPesälä Sormunen R. Lehenkari P. (2013). HIF-1α Is Upregulated in Human Mesenchymal Stem Cells. Stem Cells 31 (9), 1902–1909. 10.1002/stem.1435 23744828

[B67] Pi C. Yang Y. Sun Y. Wang H. Sun H. Ma M. (2019). Nicotinamide Phosphoribosyltransferase Postpones Rat Bone Marrow Mesenchymal Stem Cell Senescence by Mediating NAD+-Sirt1 Signaling. Aging 11 (11), 3505–3522. 10.18632/aging.101993 31175267 PMC6594813

[B68] Puttagunta R. Tedeschi A. Sória M. G. Hervera A. Lindner R. Rathore K. I. (2014). PCAF-dependent Epigenetic Changes Promote Axonal Regeneration in the central Nervous System. Nat. Commun. 5, 3527. 10.1038/ncomms4527 24686445

[B69] Regan J. N. Lim J. Shi Y. Joeng K. S. Arbeit J. M. Shohet R. V. (2014). Up-regulation of Glycolytic Metabolism Is Required for HIF1 -driven Bone Formation. Proc. Natl. Acad. Sci. 111 (23), 8673–8678. 10.1073/pnas.1324290111 24912186 PMC4060724

[B70] Rendina-Ruedy E. Guntur A. R. Rosen C. J. (2017). Intracellular Lipid Droplets Support Osteoblast Function. Adipocyte 6 (3), 250–258. 10.1080/21623945.2017.1356505 28792783 PMC5638385

[B71] Rupprecht A. Sittner D. Smorodchenko A. Hilse K. E. Goyn J. Moldzio R. (2014). Uncoupling Protein 2 and 4 Expression Pattern during Stem Cell Differentiation Provides New Insight into Their Putative Function. PLoS One 9 (2), e88474. 10.1371/journal.pone.0088474 24523901 PMC3921169

[B72] Semenza G. L. (2012). Hypoxia-inducible Factors in Physiology and Medicine. Cell 148 (3), 399–408. 10.1016/j.cell.2012.01.021 22304911 PMC3437543

[B73] Shares B. H. Busch M. White N. Shum L. Eliseev R. A. (2018). Active Mitochondria Support Osteogenic Differentiation by Stimulating β-catenin Acetylation. J. Biol. Chem. 293 (41), 16019–16027. 10.1074/jbc.ra118.004102 30150300 PMC6187642

[B74] Sharma D. Yu Y. Shen L. Zhang G.-F. Karner C. M. (2021). SLC1A5 Provides Glutamine and Asparagine Necessary for Bone Development in Mice. Elife 10, e71595. 10.7554/elife.71595 34647520 PMC8553342

[B75] Shen G. Zhang H. Jia P. Li G. Wang X. Zhou X. (2018). GOLM1 Stimulation of Glutamine Metabolism Promotes Osteoporosis via Inhibiting Osteogenic Differentiation of BMSCs. Cell Physiol Biochem 50 (5), 1916–1928. 10.1159/000494872 30396165

[B76] Shen L. Sharma D. Yu Y. Long F. Karner C. M. (2021). Biphasic Regulation of Glutamine Consumption by WNT during Osteoblast Differentiation. J. Cell Sci 134 (1), jcs251645. 10.1242/jcs.251645 33262314 PMC7823158

[B77] Shi Y. Chen J. Karner C. M. Long F. (2015). Hedgehog Signaling Activates a Positive Feedback Mechanism Involving Insulin-like Growth Factors to Induce Osteoblast Differentiation. Proc. Natl. Acad. Sci. USA 112, 4678–4683. 10.1073/pnas.1502301112 25825734 PMC4403181

[B78] Shum L. C. White N. S. Mills B. N. de Mesy Bentley K. L. Eliseev R. A. (2016). Energy Metabolism in Mesenchymal Stem Cells during Osteogenic Differentiation. Stem Cells Dev. 25 (2), 114–122. 10.1089/scd.2015.0193 26487485 PMC4733323

[B79] Shyh-Chang N. Daley G. Q. (2015). Metabolic Switches Linked to Pluripotency and Embryonic Stem Cell Differentiation. Cell Metab. 21 (3), 349–350. 10.1016/j.cmet.2015.02.011 25738450

[B80] Singh K. Krug L. Basu A. Meyer P. Treiber N. Vander Beken S. (2017). Alpha-Ketoglutarate Curbs Differentiation and Induces Cell Death in Mesenchymal Stromal Precursors with Mitochondrial Dysfunction. Stem Cells 35 (7), 1704–1718. 10.1002/stem.2629 28398002

[B81] Smith C. O. Eliseev R. A. (2021). Energy Metabolism during Osteogenic Differentiation: The Role of Akt. Stem Cells Dev. 30 (3), 149–162. 10.1089/scd.2020.0141 33307974 PMC7876359

[B82] Song J. Li J. Yang F. Ning G. Zhen L. Wu L. (2019). Nicotinamide Mononucleotide Promotes Osteogenesis and Reduces Adipogenesis by Regulating Mesenchymal Stromal Cells via the SIRT1 Pathway in Aged Bone Marrow. Cell Death Dis 10 (5), 336. 10.1038/s41419-019-1569-2 31000692 PMC6472410

[B83] Stegen S. van Gastel N. Eelen G. Ghesquière B. D’Anna F. Thienpont B. (2016). HIF-1α Promotes Glutamine-Mediated Redox Homeostasis and Glycogen-dependent Bioenergetics to Support Postimplantation Bone Cell Survival. Cell Metab. 23 (2), 265–279. 10.1016/j.cmet.2016.01.002 26863487 PMC7611069

[B84] Su X. Wellen K. E. Rabinowitz J. D. (2016). Metabolic Control of Methylation and Acetylation. Curr. Opin. Chem. Biol. 30, 52–60. 10.1016/j.cbpa.2015.10.030 26629854 PMC4731252

[B85] Suchacki K. J. Cawthorn W. P. Rosen C. J. (2016). Bone Marrow Adipose Tissue: Formation, Function and Regulation. Curr. Opin. Pharmacol. 28, 50–56. 10.1016/j.coph.2016.03.001 27022859 PMC5351553

[B86] Sun C. Shang J. Yao Y. Yin X. Liu M. Liu H. (2016). O‐Glc NA Cylation: a Bridge between Glucose and Cell Differentiation. J. Cell. Mol. Med. 20 (5), 769–781. 10.1111/jcmm.12807 26929182 PMC4831356

[B87] Sun W. Shi Y. Lee W.-C. Lee S.-Y. Long F. (2016). Rictor Is Required for Optimal Bone Accrual in Response to Anti-sclerostin Therapy in the Mouse. Bone 85, 1–8. 10.1016/j.bone.2016.01.013 26780446 PMC4896354

[B88] Tan H. Y. Eskandari R. Shen D. Zhu Y. Liu T.-W. Willems L. I. (2018). Direct One-step Fluorescent Labeling of O-GlcNAc-Modified Proteins in Live Cells Using Metabolic Intermediates. J. Am. Chem. Soc. 140 (45), 15300–15308. 10.1021/jacs.8b08260 30296064

[B89] Tan S. H. Senarath-Yapa K. Chung M. T. Longaker M. T. Wu J. Y. Nusse R. (2014). Wnts Produced by Osterix-Expressing Osteolineage Cells Regulate Their Proliferation and Differentiation. Proc. Natl. Acad. Sci. USA 111 (49), E5262–E5271. 10.1073/pnas.1420463111 25422448 PMC4267375

[B90] Tang X. Ma S. Li Y. Sun Y. Zhang K. Zhou Q. (2020). Evaluating the Activity of Sodium Butyrate to Prevent Osteoporosis in Rats by Promoting Osteal GSK-3β/Nrf2 Signaling and Mitochondrial Function. J. Agric. Food Chem. 68 (24), 6588–6603. 10.1021/acs.jafc.0c01820 32459091

[B91] Teperino R. Amann S. Bayer M. McGee S. L. Loipetzberger A. Connor T. (2012). Hedgehog Partial Agonism Drives Warburg-like Metabolism in Muscle and Brown Fat. Cell 151, 414–426. 10.1016/j.cell.2012.09.021 23063129

[B92] Tischler J. Gruhn W. H. Reid J. Allgeyer E. Buettner F. Marr C. (2019). Metabolic Regulation of Pluripotency and Germ Cell Fate through α-ketoglutarate. EMBO J. 38 (1), e99518. 10.15252/embj.201899518 30257965 PMC6315289

[B93] Tormos K. V. Chandel N. S. (2010). Inter-connection between Mitochondria and HIFs. J. Cell Mol Med 14 (4), 795–804. 10.1111/j.1582-4934.2010.01031.x 20158574 PMC2987233

[B94] Wang T. He H. Liu S. Jia C. Fan Z. Zhong C. (2019). Autophagy: A Promising Target for Age-Related Osteoporosis. Cdt 20 (3), 354–365. 10.2174/1389450119666180626120852 29943700

[B95] Wang W. Zhang Y. Lu W. Liu K. (2015). Mitochondrial Reactive Oxygen Species Regulate Adipocyte Differentiation of Mesenchymal Stem Cells in Hematopoietic Stress Induced by Arabinosylcytosine. PLoS One 10 (3), e0120629. 10.1371/journal.pone.0120629 25768922 PMC4359087

[B96] Wang Y. Nishida S. Boudignon B. M. Burghardt A. Elalieh H. Z. Hamilton M. M. (2007). IGF-I Receptor Is Required for the Anabolic Actions of Parathyroid Hormone on Bone. J. Bone Miner Res. 22 (9), 1329–1337. 10.1359/jbmr.070517 17539737 PMC10702248

[B97] Wellen K. E. Thompson C. B. (2012). A Two-Way Street: Reciprocal Regulation of Metabolism and Signalling. Nat. Rev. Mol. Cell Biol 13 (4), 270–276. 10.1038/nrm3305 22395772

[B98] Wu G.-J. Chen J.-T. Lin P.-I. Cherng Y.-G. Yang S.-T. Chen R.-M. (2020). Inhibition of the Estrogen Receptor Alpha Signaling Delays Bone Regeneration and Alters Osteoblast Maturation, Energy Metabolism, and Angiogenesis. Life Sci. 258, 118195. 10.1016/j.lfs.2020.118195 32781073

[B99] Yang J. Ueharu H. Mishina Y. (2020). Energy Metabolism: A Newly Emerging Target of BMP Signaling in Bone Homeostasis. Bone 138, 115467. 10.1016/j.bone.2020.115467 32512164 PMC7423769

[B100] Yang X. Qian K. (2017). Protein O-GlcNAcylation: Emerging Mechanisms and Functions. Nat. Rev. Mol. Cell Biol 18 (7), 452–465. 10.1038/nrm.2017.22 28488703 PMC5667541

[B101] Ye C. Tu B. P. (2018). Sink into the Epigenome: Histones as Repositories that Influence Cellular Metabolism. Trends Endocrinol. Metab. 29 (9), 626–637. 10.1016/j.tem.2018.06.002 30001904 PMC6109460

[B102] Zanotti S. Canalis E. (2017). Parathyroid Hormone Inhibits Notch Signaling in Osteoblasts and Osteocytes. Bone 103, 159–167. 10.1016/j.bone.2017.06.027 28676438 PMC5568480

[B103] Zanotti S. Yu J. Sanjay A. Schilling L. Schoenherr C. Economides A. N. (2017). Sustained Notch2 Signaling in Osteoblasts, but Not in Osteoclasts, Is Linked to Osteopenia in a Mouse Model of Hajdu-Cheney Syndrome. J. Biol. Chem. 292 (29), 12232–12244. 10.1074/jbc.m117.786129 28592489 PMC5519372

[B104] Zhang T. Tian T. Lin Y. (2021). Functionalizing Framework Nucleic Acid-Based Nanostructures for Biomedical Application. Adv. Mater. 2021, e2107820. 10.1002/adma.202107820 34787933

[B105] Zhang Y. Yang J.-H. (2013). Activation of the PI3K/Akt Pathway by Oxidative Stress Mediates High Glucose-Induced Increase of Adipogenic Differentiation in Primary Rat Osteoblasts. J. Cell. Biochem. 114 (11), 2595–2602. 10.1002/jcb.24607 23757055

[B106] Żurek A. Mizerska-Kowalska M. Sławińska-Brych A. Kaławaj K. Bojarska-Junak A. Kandefer-Szerszeń M. (2019). Alpha Ketoglutarate Exerts a Pro-osteogenic Effect in Osteoblast Cell Lines through Activation of JNK and mTOR/S6K1/S6 Signaling Pathways. Toxicol. Appl. Pharmacol. 374, 53–64. 10.1016/j.taap.2019.04.024 31051157

